# Potential Utility of Combined Salivary Calprotectin and Anti-Cyclic Citrullinated Peptide in Rheumatoid Arthritis Assessment

**DOI:** 10.3390/diagnostics16010023

**Published:** 2025-12-21

**Authors:** Misong Kim, Young Il Kim, Yeon-Ah Lee, Seung-Jae Hong

**Affiliations:** 1 Division of Rheumatology, Department of Internal Medicine, Kyung Hee University College of Medicine, Kyung Hee University Medical Center, Seoul 02447, Republic of Korea; 2 Department of Biomedical Science, Graduate School, Kyung Hee University, Seoul 02447, Republic of Korea; 3 Medical Science Research Institute, Kyung Hee University Medical Center, Seoul 02447, Republic of Korea

**Keywords:** anti-CCP, calprotectin, rheumatoid arthritis, salivary biomarker

## Abstract

**Background/Objectives**: Rheumatoid arthritis (RA) is a chronic autoimmune disease characterized by persistent synovial inflammation and progressive joint damage. Although serum biomarkers such as rheumatoid factor (RF) and anti-cyclic citrullinated peptide (anti-CCP) are widely used, blood-based testing is invasive. Saliva has emerged as a noninvasive diagnostic medium with clinical potential. This study aimed to evaluate the potential utility of salivary calprotectin and anti-CCP antibodies for discriminating patients with RA from healthy controls. **Methods**: Saliva samples were collected from 58 RA patients and 50 healthy controls. Salivary calprotectin and anti-CCP antibody levels were quantified using enzyme-linked immunosorbent assay. The diagnostic performance was evaluated using receiver operating characteristic curve analysis and logistic regression models that incorporated both biomarkers and clinical variables. **Results**: Patients with RA exhibited significantly higher salivary calprotectin and anti-CCP levels than controls (both *p* < 0.001). Calprotectin showed high sensitivity (79.31%), whereas anti-CCP displayed high specificity (84.00%). Salivary calprotectin was associated with disease duration and joint damage, while anti-CCP correlated with the erythrocyte sedimentation rate, RF, and serum anti-CCP. A multivariate model combining salivary biomarkers with clinical factors indicated an excellent diagnostic discrimination. **Conclusions**: Salivary calprotectin and anti-CCP antibodies show potential as complementary noninvasive biomarkers for distinguishing patients with established RA from healthy controls. However, as saliva samples were not collected at the time of initial diagnosis, these findings primarily support disease discrimination rather than early detection. Further prospective studies involving newly diagnosed and at-risk populations are required to clarify their role in early diagnosis, monitoring, and clinical implementation.

## 1. Introduction

Rheumatoid arthritis (RA) is a chronic inflammatory arthritis affecting 0.5–1% of the global population. This condition is characterized by persistent synovitis, which leads to joint cartilage damage, bone erosion, and progressive disability [1,2]. Early diagnosis and active treatment are crucial for achieving remission and preventing irreversible joint deformity [3]. Current disease activity assessments rely primarily on clinical indicators, such as the Clinical Disease Activity Index (CDAI), Simplified Disease Activity Index (SDAI), and Disease Activity Score in 28 joints (DAS28), which are based on subjective evaluations and may not accurately reflect underlying inflammatory processes [4,5]. Acute-phase reactants such as C-reactive protein (CRP) and erythrocyte sedimentation rate (ESR) are commonly used as indicators of systemic inflammation. However, these non-specific markers can remain elevated even in patients with clinically low disease activity, highlighting the urgent need for objective and easily accessible biomarkers [6].

Hence, novel biomarkers that objectively reflect inflammatory activity are required. Calprotectin, a complex of the S100A8 and S100A9 proteins predominantly present in the neutrophil cytoplasm, is released upon inflammatory stimulation and has emerged as a promising inflammatory marker [7]. This protein has been extensively validated in inflammatory bowel disease and various systemic rheumatic diseases, including psoriatic arthritis, ankylosing spondylitis, and lupus [8,9,10,11]. Importantly, elevated calprotectin levels have been reported in RA synovial tissues, and salivary calprotectin levels are significantly higher in patients with Sjögren’s syndrome (SS) than in healthy controls [12,13]. Considering the presence of calprotectin in various bodily fluids and its established utility in multiple inflammatory conditions. Based on this evidence, we hypothesized that salivary calprotectin could serve as an objective biomarker for the assessment of RA.

In addition to novel inflammatory markers, established RA biomarkers may also be useful for salivary-based diagnosis. Anti-cyclic citrullinated peptide (anti-CCP) antibodies are well-established biomarkers for both the diagnosis and prognosis of RA [14,15,16,17]. Recently, the diagnostic potential of salivary immunoglobulin A (IgA) anti-CCP antibodies has garnered attention, although the findings remain inconsistent [18]. Earlier studies have reported that patients positive for salivary IgA anti-CCP levels have significantly lower rates of joint erosion over a 6-year period [19]. In contrast, more recent evidence suggests a positive association between salivary IgA anti-CCP and elevated disease activity [20]. Nevertheless, salivary anti-CCP levels correlate with serum anti-CCP concentrations in patients with RA, supporting their potential as convenient and noninvasive diagnostic markers [21].

Given the potential of both calprotectin and anti-CCP as RA biomarkers, their application in salivary diagnosis has become particularly attractive, considering the limitations of current blood-based assessment methods. Blood-based biomarker assessment has several limitations such as the need for invasive procedures, patient discomfort, potential infection risk, and additional healthcare costs [22]. Salivary biomarkers offer compelling advantages as noninvasive, painless, and cost-effective alternatives that can be collected repeatedly without specialized personnel [23]. The molecular connectivity between blood and saliva, facilitated by capillary networks surrounding the salivary glands, enables blood-derived molecules to alter the salivary biochemical composition, making oral fluid a potential reservoir of disease-related molecular information [24].

This study aimed to investigate the potential utility of salivary calprotectin and anti-CCP antibodies for discriminating patients with RA from healthy controls and for exploring their association with disease-related features. Our findings may contribute to the development of accessible, noninvasive diagnostic tools that can enhance RA management in clinical practice.

## 2. Materials and Methods

### 2.1. Study Design and Participants

This prospective observational study was conducted at Kyung Hee University Hospital between 12 October 2021 and 8 January 2024. A total of 58 patients with RA were enrolled in this study. The inclusion criteria for RA patients were a diagnosis of RA based on the 1987 American College of Rheumatology (ACR) or the 2010 ACR/European Alliance of Associations for Rheumatology (EULAR) classification criteria. In addition, 50 HCs were recruited from individuals undergoing routine health examinations at the same institution during the study period. HCs were not randomly sampled from the general population but were consecutively enrolled based on predefined inclusion and exclusion criteria. To minimize selection bias, controls were age- and sex-matched to patients with RA and were screened to exclude inflammatory, autoimmune, or musculoskeletal diseases, as well as the use of anti-inflammatory medications. This approach was intended to ensure comparability between groups while reflecting a clinically relevant reference population. Demographic information, clinical characteristics, and laboratory data—including DAS28, ESR, and CRP, rheumatoid factor (RF), and anti-CCP levels—were collected at enrollment.

### 2.2. Ethical Statement

This study was conducted in accordance with the Declaration of Helsinki and was approved by the Institutional Review Board of Kyung Hee University Medical Center (KHMC 2021-08-074; approved on 8 October 2021). Written informed consent was obtained from all subjects involved in the study.

### 2.3. Saliva Collection

Saliva samples were collected during scheduled clinical visits, without requiring a predefined disease activity state. To prevent saliva contamination, the participants were instructed to refrain from eating, drinking, smoking, or performing oral hygiene procedures for at least 30 min prior to saliva collection. Before spitting, the participants rinsed their mouths with water, and saliva was collected using the unstimulated whole saliva method. The participants spit into a conical tube once saliva naturally accumulated in their mouth. The tubes were stored on ice to prevent protein degradation. A protein inhibitor cocktail was added, and the mixture was centrifuged at 3440 rpm for 15 min at 4 °C. The supernatant was stored at −80 °C until further analysis.

### 2.4. Salivary Calprotectin and Anti-CCP Testing

Calprotectin levels in saliva were analyzed using a Human S100A8/S100A9 Heterodimer enzyme-linked immunosorbent assay (ELISA) kit (# DS8900; R&D Systems, Minneapolis, MN, USA), and anti-CCP in saliva was measured using a Quanta Lite CCP 3.1 IgG/IgA ELISA kit (#704550; Werfen, Bedford, MA, USA).

### 2.5. Statistical Analysis

Continuous variables were tested for normality using the Shapiro–Wilk test. Normally distributed variables are presented as means ± standard deviation (SD) and were compared using an independent t-test. Non-normally distributed variables are presented as medians and interquartile ranges (IQRs) and were compared using the Mann–Whitney U test. Categorical variables were analyzed using the chi-square test. Correlations between variables were examined using Spearman’s correlation test. Receiver operating characteristic (ROC) curve analysis was conducted to evaluate the diagnostic performance of salivary calprotectin and anti-CCP antibodies, including the area under the curve (AUC), sensitivity, specificity, and optimal cut-off values. Logistic regression models were constructed to compare the different combinations of biomarkers and clinical factors. The following models were tested.

- Model 1 (unadjusted): salivary calprotectin and salivary anti-CCP only;
- Model 2 (partially adjusted): Model 1 + sex, age, and body mass index (BMI);
- Model 3 (clinically refined): salivary calprotectin, salivary anti-CCP, age, sex, alcohol consumption, dry mouth symptoms, and hyperlipidemia.

The AUCs of the three models were compared to determine whether combining the two salivary biomarkers and adjusting for clinical confounders improved the discriminatory power. The statistical significance level was set at *p* < 0.05, and all analyses were performed using the Statistical Package for the Social Sciences (v29.0) and GraphPad Prism (v8.0).

### 2.6. Artificial Intelligence Tool Usage

Google Gemini 3 was utilized for the purpose of linguistic refinement. This tool was employed to check grammar, structure, spelling, punctuation and formatting to improve the clarity and readability of the text. It was not used to generate any scientific content.

## 3. Results

### 3.1. Demographic and Clinical Characteristics

A total of 58 patients with RA and 50 HCs were included in this study. The demographic and clinical characteristics of the patients are shown in Table 1.

No statistically significant differences in age, sex, BMI, or smoking status were observed between patients with RA and HCs. Alcohol consumption was lower (*p* = 0.030) and dry mouth symptoms were more common (*p* = 0.003) in the RA group than in the HCs. Hyperlipidemia was significantly more prevalent in the RA group than in HCs (*p* < 0.001). Most patients with RA were on conventional disease-modifying antirheumatic drugs (93.1%), and 62.1% received glucocorticoids.

### 3.2. Salivary Calprotectin and Anti-CCP Levels in the RA Group and HCs

**Figure 1:**
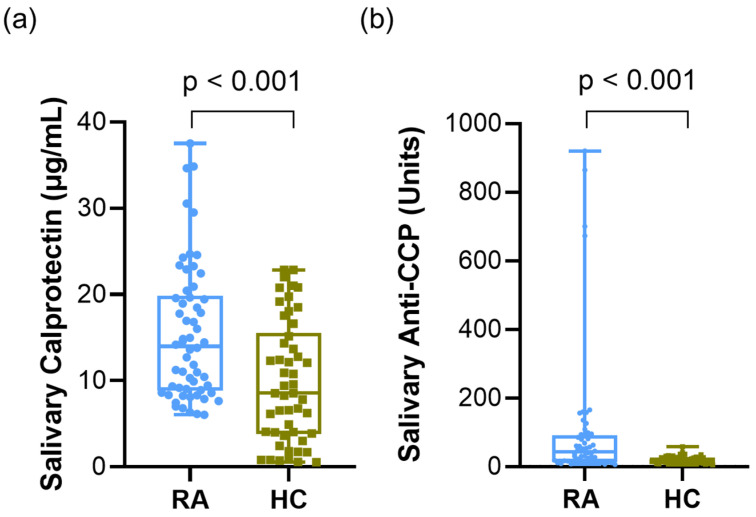
Salivary calprotectin and anti-CCP levels in patients with RA and HCs. (**a**) Salivary calprotectin levels in RA vs. HC. (**b**) Salivary anti-CCP levels in RA vs. HC. Data were analyzed using the Mann–Whitney U test. Abbreviations: CCP, cyclic citrullinated peptide; RA, rheumatoid arthritis; HCs, healthy controls. Statistical significance: *p* < 0.05.

Patients with RA had significantly higher salivary calprotectin and anti-CCP levels than the HCs. For salivary calprotectin, the RA group showed higher levels [mean ± SD, 15.45 ± 7.87 µg/mL; median (IQR), 13.99 (11.02) µg/mL] than the HC group [9.81 ± 7.06 µg/mL; 8.55 (11.68) µg/mL] (*p* < 0.001, Figure 1a). Similarly, salivary anti-CCP levels were markedly increased in the RA group [102.46 ± 195.60 units; 42.52 (77.04) units] compared to that in the HC group [20.05 ± 10.28 units; 18.7 (14.26) units] (*p* < 0.001, Figure 1b). Because the data were not normally distributed, the Mann–Whitney U test was used for group comparisons.

### 3.3. Association of Salivary Biomarkers with Radiographic Joint Damage in Patients with RA

**Figure 2:**
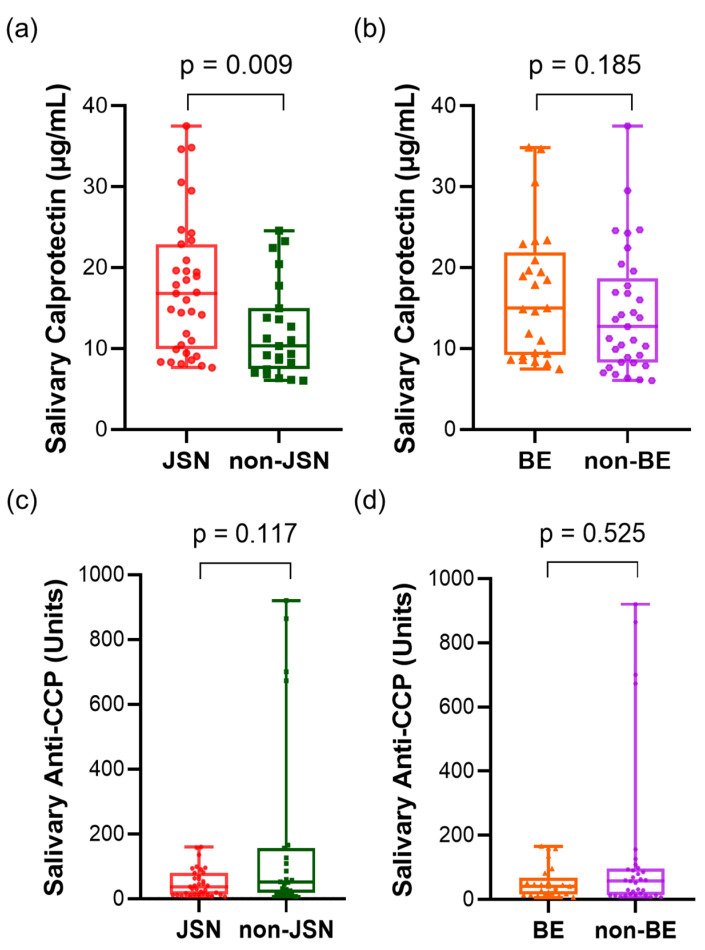
Salivary calprotectin and anti-CCP concentrations in patients with RA according to radiographic joint damage. (**a**) Salivary calprotectin levels in JSN vs. non-JSN groups. (**b**) Salivary calprotectin levels in BE vs. non-BE groups. (**c**) Salivary anti-CCP levels in JSN vs. non-JSN groups. (**d**) Salivary anti-CCP levels in BE vs. non-BE groups. Statistical analysis was performed using the Mann–Whitney U test. Abbreviations: CCP, cyclic citrullinated peptide; JSN, joint space narrowing; BE, bone erosion. Statistical significance: *p* < 0.05.

In 58 patients with RA, radiographic examination of the hands and feet revealed that 35 of the 58 patients (60.3%) had visible joint space narrowing (JSN) and 25 (43.1%) had visible bone erosion (BE). In the RA group, those with JSN had higher calprotectin levels than those without JSN [JSN group, median (IQR), 16.83 (13.01) µg/mL; non-JSN group, median (IQR), 10.31 (7.54) µg/mL; *p* = 0.009, Figure 2a]. Although the difference was not statistically significant, salivary calprotectin levels were higher in the BE group than in the non-erosion group [15.00 (12.72) vs. 12.70 (10.42) µg/mL; *p* = 0.185, Figure 2b]. Salivary anti-CCP levels were lower in patients without JSN [JSN vs. non-JSN; 36.68 (66.34) vs. 51.70 (136.89) units; *p* = 0.117, Figure 2c] and without BE [BE vs. non-BE; 40.16 (51.60) vs. 56.90 (82.53) units; *p* = 0.525, Figure 2d], although the differences were not statistically significant. To further evaluate the association with combined structural damage, we stratified the patients into four groups based on the presence of JSN and BE (Table S1). Although the difference did not reach statistical significance (*p* = 0.071), a clear trend was observed in which salivary calprotectin levels increased with the severity of joint damage. The mean levels rose from 12.04 ± 5.78 µg/mL in patients with neither JSN nor BE to 17.87 ± 8.36 µg/mL in those with both JSN and BE.

### 3.4. Correlation Between Salivary Biomarkers and Clinical Parameters

Spearman’s correlation analysis was conducted to examine the association between salivary calprotectin and anti-CCP levels and clinical variables in patients with RA (Table 2).

Salivary calprotectin levels significantly and positively correlated with disease duration (r = 0.274, *p* = 0.038). No other clinical variables showed significant correlations with salivary calprotectin levels. In contrast, salivary anti-CCP levels were significantly correlated with several serological markers. Specifically, positive correlations were observed between ESR (r = 0.311, *p* = 0.017), RF (r = 0.516, *p* < 0.001), and serum anti-CCP (r = 0.699, *p* < 0.001). To further assess the agreement between serum and salivary anti-CCP levels, Bland–Altman analysis was performed, revealing a systematic bias (mean difference = 1.09) but consistent agreement, with 95% limits ranging from −1.47 to 3.65 (Figure S1). No significant correlation was identified between salivary calprotectin and anti-CCP levels (r = −0.047, *p* = 0.724). Furthermore, no significant interaction was observed between the two biomarkers in logistic regression models (*p* = 0.584, Table S2), suggesting that they represent distinct pathological processes and contribute independently to the diagnosis of RA.

### 3.5. Diagnostic Value of Salivary Calprotectin and Anti-CCP Based on ROC-Derived Cut-Off

**Figure 3:**
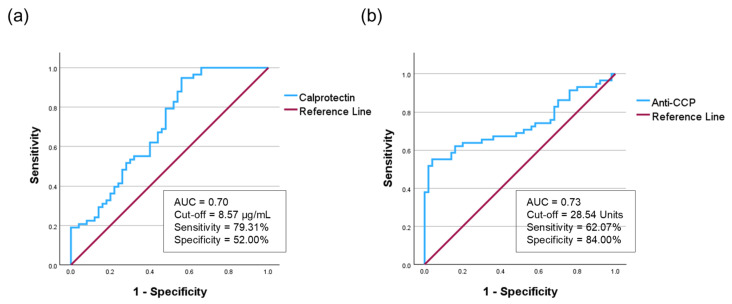
The receiver operating characteristic curve of salivary calprotectin and salivary anti-CCP. (**a**) Salivary calprotectin showed an AUC of 0.70. (**b**) Salivary anti-CCP demonstrated an AUC of 0.73. AUC, area under the curve; anti-CCP, anti-cyclic citrullinated peptide.

To evaluate the diagnostic performance of salivary biomarkers in distinguishing patients with RA from HC, ROC analyses were performed for calprotectin and anti-CCP, followed by logistic regression analysis based on the ROC-derived cut-off values (Figure 3).

For salivary calprotectin, the optimal cutoff was 8.57 µg/mL (sensitivity 79.31%, specificity 52.00%). The AUC was 0.70 (95% confidence interval CI = 0.60–0.80), with a Youden Index of 0.31, indicating fair diagnostic performance (Figure 3a, Table S3). For salivary anti-CCP, the cutoff was 28.54 units (sensitivity 62.07%, specificity 84.00%), yielding an AUC of 0.73 (95% CI: 0.64–0.83) and a Youden Index of 0.46 (Figure 3b, Table S3). Given the high variability of salivary anti-CCP levels, we further performed a sensitivity analysis using quartile-based stratification. The highest quartile (Q4) showed a marked increase in RA risk (adjusted OR = 24.50, *p* < 0.001) with an AUC of 0.86, confirming that the diagnostic accuracy was driven by high-titer associations rather than outliers (Table S4).

Based on this cutoff, logistic regression analysis demonstrated that individuals with salivary calprotectin levels above the threshold had significantly higher odds of having RA than HCs (OR = 4.153; 95% CI = 1.787–9.653; *p* < 0.001). Logistic regression analysis showed that individuals with salivary anti-CCP levels above this cutoff had significantly increased odds of developing RA (OR = 8.591; 95% CI = 3.411–21.634; *p* < 0.001) (Table 3).

Both salivary calprotectin and anti-CCP are significantly associated with RA, supporting their utility as noninvasive biomarkers. Calprotectin demonstrated higher sensitivity, whereas anti-CCP showed superior specificity and discriminative power.

### 3.6. Diagnostic Performance According to Disease Activity

Subgroup analysis based on disease activity (DAS28-ESR) confirmed that both biomarkers maintained significant diagnostic value regardless of the disease status (Table 4). 

Salivary anti-CCP showed excellent performance in the moderate/high-activity group (AUC = 0.79, *p* = 0.002). Crucially, even in the low-activity/remission group, both calprotectin (AUC = 0.71, *p* < 0.001) and anti-CCP (AUC = 0.72, *p* < 0.001) retained their discriminatory abilities, suggesting their utility even in patients with low systemic inflammation.

### 3.7. Logistic Regression and ROC Modeling for the Prediction of RA

Logistic regression analysis was performed to identify the predictive factors for RA (Table 5).

**Figure 4:**
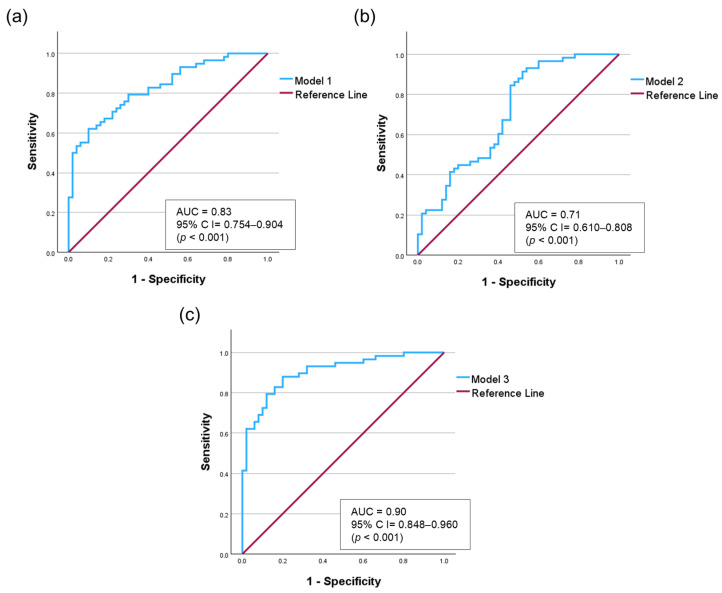
Receiver operating characteristic (ROC) curves comparing three logistic regression models. (**a**) Model 1: Salivary biomarkers (calprotectin and anti-CCP). (**b**) Model 2: Model 1 adjusted for demographic factors (age, sex, and BMI). (**c**) Model 3: Model 1 adjusted for demographic and clinical factors (age, sex, alcohol consumption, dry mouth, and hyperlipidemia.

Salivary calprotectin (OR = 1.111, 95% CI = 1.047–1.179, *p* = 0.001) and salivary anti-CCP (OR = 1.049, 95% CI = 1.022–1.077, *p* = 0.001) were significantly associated with RA presence. Additionally, dry mouth symptoms (OR = 5.968, 95% CI = 1.624–21.931, *p* = 0.007), alcohol consumption (*p* = 0.031), and hyperlipidemia (*p* = 0.001) were statistically significant. To assess the diagnostic performance of the predictive factors, an ROC curve analysis was conducted using three logistic regression models (Figure 4).

Model 1, including only salivary biomarkers (salivary calprotectin and salivary anti-CCP), showed an AUC of 0.83 (95% CI = 0.754–0.904). Model 2, adjusted for demographic factors (age, sex, and BMI), yielded an AUC of 0.71 (95% CI = 0.610–0.808). We confirmed that this decrease in the AUC was not due to multicollinearity, as the variance inflation factor (VIF) values for all variables were less than 6 (Table S5). Model 3, which incorporated salivary calprotectin, salivary anti-CCP, age, sex, alcohol consumption, dry mouth symptoms, and hyperlipidemia, exhibited the best diagnostic accuracy, achieving an AUC of 0.90 (95% CI = 0.848–0.960, *p* < 0.001). To validate model stability and address potential overfitting, we performed bootstrapping (1000 iterations), confirming the reliability of Model 3 with a mean AUC of 0.91 (Table S6). To further ensure the stability of our diagnostic model against potential confounders, we performed comprehensive sensitivity analyses (Tables S7–S10). Regarding demographic factors, hyperlipidemia showed no significant interaction with either biomarker (*p* > 0.05). Although salivary anti-CCP showed an interaction with alcohol consumption (*p* = 0.026), it remained a predictor in nondrinkers (OR = 1.09, *p* = 0.001), who comprised the majority of the cohort. Stratification according to dry mouth symptoms revealed no significant interaction (*p* = 0.669). Notably, salivary calprotectin level remained a significant predictor of normal salivary function (adjusted OR = 1.12, *p* = 0.002). Furthermore, the diagnostic performance was strengthened in the age-matched analysis (calprotectin: adjusted OR = 1.55, AUC = 0.93; anti-CCP: adjusted OR = 1.07, AUC = 0.87). Finally, even after adjusting for ESR, salivary calprotectin remained a significant independent predictor (adjusted OR = 1.35, *p* = 0.008), confirming that it captures local inflammation, distinct from systemic markers.

Thus, combining salivary biomarkers with selected clinical variables could enhance model performance, and refined variable selection could further improve the diagnostic accuracy for RA.

## 4. Discussion

Salivary calprotectin and anti-CCP antibodies may serve as complementary noninvasive biomarkers for distinguishing patients with RA from healthy controls. In this study, both salivary biomarkers were significantly elevated in patients with established RA compared to controls, and their combined use was associated with improved diagnostic discrimination when combined with selected clinical variables. These findings are consistent with previous reports suggesting that salivary biomarkers reflect systemic and local inflammatory processes in RA [21,25]. However, it should be emphasized that saliva samples in this study were collected during routine clinical visits rather than at the time of initial diagnosis. Therefore, the observed diagnostic performance should be interpreted as reflecting the ability to differentiate established RA from controls, rather than confirming utility for early or incident RA detection.

Salivary calprotectin demonstrated relatively high sensitivity for RA discrimination, which is consistent with its role as a marker of neutrophil-driven inflammation [26]. The observed association with disease duration and radiographic joint space narrowing suggests a possible relationship with cumulative inflammatory burden and structural damage [27], although causal inference cannot be established in this cross-sectional design. Importantly, salivary calprotectin remained independently associated with RA even after adjustment for ESR, indicating that it may capture inflammatory processes not fully reflected by systemic markers. Nevertheless, given that samples were not obtained at diagnosis, whether salivary calprotectin can reliably identify early or preclinical RA requires further prospective investigation.

Calprotectin is also a well-established biomarker for inflammatory bowel disease; however, confounding by gastrointestinal (GI) inflammation appears to be minimal [28,29]. Previous studies indicate that salivary calprotectin levels do not correlate with fecal calprotectin concentrations or indices of intestinal disease activity [30,31].

Salivary anti-CCP antibodies exhibited higher specificity and showed strong correlations with established serological markers, including serum anti-CCP and RF. The agreement between salivary and serum anti-CCP supports the biological plausibility of saliva as a surrogate medium for systemic autoimmunity assessment [32,33,34]. However, the clinical implications of salivary anti-CCP levels, particularly in relation to radiographic damage, remain incompletely understood. Interestingly, while salivary anti-CCP correlated with disease activity markers, it showed a trend toward lower radiographic damage. This paradox may reflect distinct isotype functions. Serum anti-CCP is predominantly of the IgG isotype, which is highly pathogenic, and is a strong predictor of bone erosion [35,36]. In contrast, salivary anti-CCP is predominantly secretory IgA [18,19,20]. Mucosal IgA facilitates immune exclusion, potentially sequestering antigens and limiting the switch to destructive IgG. Thus, a robust salivary IgA response may serve a protective regulatory role, mitigating structural damage despite high systemic inflammation [37].

The lack of correlation between salivary calprotectin and anti-CCP suggests that these biomarkers reflect distinct inflammatory pathways, with calprotectin primarily indicating neutrophil-mediated acute inflammation [38], whereas anti-CCP represents adaptive autoimmunity [39]. This biological distinction may explain their complementary diagnostic contribution. Subgroup analyses indicated that both biomarkers retained discriminatory ability even in patients with low disease activity or remission. However, because this cohort consisted predominantly of patients with established RA, these results do not confirm their performance at disease onset. Future studies incorporating newly diagnosed and at-risk individuals are necessary to clarify their role in early RA detection and screening.

The significant association between dry mouth symptoms and RA observed in our multivariate model (OR = 5.968) necessitates the consideration of secondary SS as a potential comorbidity influencing biomarker levels. Although salivary biomarkers theoretically offer the potential to detect glandular inflammation, their clinical utility in secondary SS is often limited by pre-analytical challenges [40]. Hyposalivation can hinder adequate sample collection and standardization, whereas periodontal disease, frequently exacerbated by xerostomia, may act as a confounding factor by elevating inflammatory markers [41]. Therefore, when applying salivary testing to patients with RA and secondary SS, rigorous standardization and careful interpretation of oral health status are essential. However, our stratified analysis suggests that the absence of statistical significance in the dry mouth subgroup may have been influenced by the limited number of controls (*n* = 3), given that the adjusted odds ratio was higher than in the non-dry mouth group (1.39 vs. 1.12). Furthermore, the robust association confirmed in participants with normal salivary function demonstrates that this discriminatory ability is not merely an artifact of hyposalivation.

Our findings align with the mucosal origin hypothesis of RA [42]. Recent studies have highlighted that total serum IgA and IgA-class autoantibodies are elevated in adult patients with RA and juvenile idiopathic arthritis (JIA), suggesting a shared mucosal immune dysregulation. Integrating systemic IgA markers with salivary profiling may provide a more comprehensive view of the mucosal-systemic axis [43,44]. Moreover, non-invasive saliva collection is particularly advantageous for pediatric JIA populations, offering a child-friendly alternative to repeated venipuncture for longitudinal monitoring.

This study had some limitations. First, the study was conducted in a single-center cohort with a relatively small sample size, which may limit the generalizability of our findings. However, internal validation using bootstrapping confirmed the stability of our diagnostic model (mean AUC = 0.91), mitigating concerns about potential overfitting due to the limited sample size. Multicenter validation studies are necessary to further confirm the robustness of these results. Second, saliva samples were not collected at the time of initial diagnosis but rather from patients with established RA (mean disease duration, 13.2 years). Although the significant difference between RA patients and healthy controls supports their potential as surrogate markers for RA, the absence of data from incident cases limits the ability to definitively confirm their utility for early diagnosis. Third, the cross-sectional design prevented causal analysis of biomarker changes over time. Notably, we observed no significant differences in biomarker levels across the medication subgroups (Table S11), suggesting that concurrent treatments did not confound the discriminatory ability in this study. Nevertheless, future longitudinal research should explore the dynamic effects of concomitant medications, oral health status (e.g., periodontal scores), and circadian variations in biomarker concentrations to further validate their clinical utility [45].

## 5. Conclusions

Salivary calprotectin and anti-CCP antibodies demonstrate potential as noninvasive surrogate biomarkers for distinguishing patients with rheumatoid arthritis from healthy controls. Their complementary characteristics—higher sensitivity for calprotectin and higher specificity for anti-CCP—were associated with improved diagnostic discrimination when combined with selected clinical variables. However, as saliva samples in this study were obtained from patients with established RA rather than at the time of diagnosis, the findings primarily support their utility in disease discrimination rather than early detection. Prospective studies incorporating incident RA cases and longitudinal sampling are required to clarify their potential role in disease discrimination and longitudinal monitoring in clinical practice.

## Figures and Tables

**Table 1:** Demographic and clinical characteristics of participants.

	RA (*n* = 58)	HC (*n* = 50)	*p*-Value
Demographic parameters			
Sex, female/male	49/9	40/10	0.542
Age, years	63.1 ± 10.6	59.6 ± 8.6	0.061
BMI, kg/m^2^	24.0 ± 3.4	24.3 ± 3.8	0.721
Disease duration, years	13.2 ± 7.7	-	-
Alcohol consumption	15 (25.9)	23 (46.0)	0.030
Smoking status	6 (10.3)	3 (6.0)	0.500
Clinical and serological parameters			
RF (IU/mL)	122.5 ± 239.9	-	-
Anti-CCP (units/mL)	438.1 ± 630.8	-	-
ESR (mm/h)	22.1 ± 18.8	-	-
CRP (mg/dL)	0.62 ± 0.64	-	-
DAS28-ESR	2.60 ± 0.85	-	-
DAS28-CRP	1.80 ± 0.64	-	-
Salivary Flow (5 min, mL)	2.8 ± 2.1	3.1 ± 1.3	0.381
Dry mouth symptoms	16 (27.6)	3 (6.0)	0.003
Radiographic images			
Joint space narrowing	35 (60.3)	-	-
Bone erosion	25 (43.1)	-	-
Comorbidities			
HTN	28 (48.3)	18 (36.0)	0.198
Hyperlipidemia	31 (73.8)	11 (22.0)	<0.001
DM	6 (10.3)	4 (8.0)	0.749
Concomitant medications			
cDMARDs	54 (93.1)	-	-
bDMARDs or tsDMARDs	12 (20.7)	-	-
Glucocorticoids	36 (62.1)	-	-
NSAIDs	33 (56.9)	-	-

Abbreviations: RA, rheumatoid arthritis; HC, healthy control; RF, rheumatoid factor; anti-CCP, anti-cyclic citrullinated peptide; BMI, body mass index; ESR, erythrocyte sedimentation rate; CRP, C-reactive protein; DAS28, Disease Activity Score in 28 Joints; DM, diabetes mellitus; HTN, hypertension; cDMARDs, conventional disease-modifying antirheumatic drugs; bDMARDs, biologic DMARDs; tsDMARDs, targeted synthetic DMARDs; NSAIDs, non-steroidal anti-inflammatory drugs.

**Table 2:** Correlation between salivary levels of calprotectin and anti-CCP and clinical data.

		Salivary Calprotectin			Salivary Anti-CCP	
	r	*p*-Value	*n*	r	*p*-Value	*n*
Age	0.044	0.740	58	0.085	0.526	58
BMI	−0.072	0.592	58	0.057	0.672	58
Disease duration	0.274	0.038	58	0.073	0.585	58
Salivary Flow (5 min, mL)	−0.177	0.184	58	−0.165	0.217	58
ESR	−0.018	0.892	58	0.311	0.017	58
CRP	−0.131	0.326	58	−0.012	0.925	58
DAS28-ESR	0.128	0.339	58	0.136	0.308	58
DAS28-CRP	0.089	0.508	58	−0.101	0.449	58
RF	0.019	0.886	58	0.516	<0.001	58
Anti-CCP	0.012	0.931	58	0.699	<0.001	58
Salivary calprotectin	1.000	-	58	−0.047	0.724	58
Salivary anti-CCP	−0.047	0.724	58	1.000	-	58

Abbreviations: ESR, erythrocyte sedimentation rate; CRP, C-reactive protein; DAS28, Disease Activity Score of 28 Joints; RF, rheumatoid factor; anti-CCP, anticyclic citrullinated peptide. Statistical significance: *p* < 0.05.

**Table 3:** Logistic regression analysis of salivary calprotectin and anti-CCP.

Variable	Cut-Off	OR	95% CI	*p*-Value
Calprotectin	8.57 µg/mL	4.153	1.787–9.653	<0.001
Anti-CCP	28.54 Units	8.591	3.411–21.634	<0.001

Abbreviations: OR, odds ratio; CI, confidence interval; anti-CCP, anti-cyclic citrullinated peptide. Statistical significance: *p* < 0.05.

**Table 4:** Diagnostic performance of salivary biomarkers stratified by RA disease activity (DAS28-ESR).

Subgroup (DAS28-ESR)	Biomarker	AUC	SE	95% CI	*p*-Value
Moderate/High (≥3.2, *n* = 12)	Salivary Calprotectin	0.68	0.079	0.524–0.836	0.054
	Salivary Anti-CCP	0.79	0.099	0.593–0.981	0.002
Low/Remission (<3.2, *n* = 46)	Salivary Calprotectin	0.71	0.053	0.602–0.809	<0.001
	Salivary Anti-CCP	0.72	0.055	0.612–0.826	<0.001

AUC, area under the curve; SE, standard error; CI, confidence interval; DAS28, Disease Activity Score of 28 joints; ESR, erythrocyte sedimentation rate. The healthy control group (*n* = 50) was used as the reference group for ROC analysis. Statistical significance: *p* < 0.05.

**Table 5:** Logistic regression analysis for factors associated with RA presence.

Variable	β	S.E.	Wald	OR	95% CI	*p*-Value
Sex	0.308	0.506	0.371	1.361	0.504–3.673	0.543
Age	0.038	0.020	3.429	1.039	0.998–1.081	0.064
BMI	−0.021	0.059	0.131	0.979	0.873–1.098	0.718
Alcohol consumption	−0.893	0.413	4.677	0.410	0.182–0.920	0.031
Dry mouth symptoms	1.786	0.664	7.238	5.968	1.624–21.931	0.007
Hyperlipidemia	1.404	0.431	10.604	4.071	1.749–9.476	0.001
Salivary calprotectin	0.105	0.030	11.947	1.111	1.047–1.179	0.001
Salivary anti-CCP	0.048	0.013	12.748	1.049	1.022–1.077	0.001

Abbreviations: β, regression coefficient; S.E., standard error; OR, odds ratio; CI, confidence interval; BMI, body mass index; anti-CCP, anti-cyclic citrullinated peptide. Statistical significance: *p* < 0.05.

## Data Availability

The data presented in this study are available in the article and Supplementary Material.

## Supplementary Materials

The following supporting information can be downloaded at: https://www.mdpi.com/article/10.3390/diagnostics16010023/s1, Figure S1: Bland–Altman plot of agreement between serum and salivary anti-CCP levels; Table S1: Salivary calprotectin levels according to the combined status of Joint Space Narrowing (JSN) and Bone Erosion (BE); Table S2: Logistic regression analysis evaluating the interaction between salivary calprotectin and anti-CCP; Table S3: Validation of diagnostic performance: Youden Index and 95% Confidence Intervals; Table S4: Logistic regression analysis and AUC of salivary Anti-CCP by quartiles; Table S5: Variance Inflation Factor (VIF) analysis for variables used in logistic regression models; Table S6: Internal validation of logistic regression models using bootstrapping (1000 iterations); Table S7: Sensitivity analysis of logistic regression models for salivary biomarkers stratified by alcohol exposure and hyperlipidemia; Table S8: Logistic regression analysis of salivary calprotectin stratified by dry mouth symptoms; Table S9: Logistic regression analysis and ROC performance in an age-matched subset; Table S10: Multivariate logistic regression analysis adjusting for Erythrocyte Sedimentation Rate (ESR) to assess the independence of salivary biomarkers; Table S11: Comparison of salivary biomarker levels in patients with RA according to medication.

## Abbreviations

The following abbreviations are used in this manuscript:

ACR	American College of Rheumatology
Anti-CCP	Anti-cyclic citrullinated peptide
AUC	Area under the curve
bDMARDs	Biologic disease-modifying antirheumatic drugs
BE	Bone erosion
BMI	Body mass index
CDAI	Clinical Disease Activity Index
cDMARDs	Conventional disease-modifying antirheumatic drugs
CI	Confidence interval
CRP	C-reactive protein
DAS28	Disease Activity Score in 28 joints
ELISA	Enzyme-linked immunosorbent assay
ESR	Erythrocyte sedimentation rate
EULAR	European Alliance of Associations for Rheumatology
HC	Healthy controls
IBD	Inflammatory bowel disease
IgA	Immunoglobulin A
IgG	Immunoglobulin G
IQR	Interquartile range
JIA	Juvenile idiopathic arthritis
JSN	Joint space narrowing
NSAIDs	Non-steroidal anti-inflammatory drugs
OR	Odds ratio
RA	Rheumatoid arthritis
RF	Rheumatoid factor
SS	Sjögren’s syndrome
