# Supplementary material for: Potential Utility of Combined Salivary Calprotectin and Anti-Cyclic Citrullinated Peptide in Rheumatoid Arthritis Assessment
Source: Diagnostics (Basel). 2025 Dec 21;16(1):23. doi: 10.3390/diagnostics16010023 (PMC12785978; doi:10.3390/diagnostics16010023)
Supplement: Supplementary file 1 [file diagnostics-16-00023-s001.zip › diagnostics-4023718-supplementary.pdf]

Supplementary Table S1. Salivary calprotectin levels according to the combined status of Joint Space Narrowing (JSN) and Bone Erosion (BE).

Group (JSN & BE Status)	n	Mean ± SD (μg/mL)	Median (IQR)	p-value*
No Damage (JSN - / BE -)	18	12.04 ± 5.78	10.68 (6.80)	0.071
Erosion Only (JSN - / BE +)	5	12.74 ± 6.57	9.35 (6.39)	
Narrowing Only (JSN + / BE -)	15	17.22 ± 8.66	16.01 (14.38)	
Combined Damage (JSN + / BE +)	20	17.87 ± 8.36	18.19 (11.71)	

*Kruskal-Wallis test

Supplementary Table S2. Logistic regression analysis evaluating the interaction between salivary calprotectin and anti-CCP.

Variable	Simple Model OR (95% CI)	p-value	Multiple Model* OR (95% CI)	p-value
Salivary Calprotectin	1.11 (1.05–1.18)	<0.001	1.14 (1.06–1.23)	<0.001
Salivary Anti-CCP	1.05 (1.02–1.08)	<0.001	1.06 (1.02–1.09)	<0.001
Interaction Term (Calprotectin × Anti-CCP)	1.00 (0.99–1.00)	0.335	1.00 (0.99–1.00)	0.584

* Adjusted for Age, Sex, Alcohol, Dry mouth, Hyperlipidemia

Supplementary Table S3. Validation of diagnostic performance: Youden Index and 95% Confidence Intervals.

Biomarker	Threshold	Sensitivity	Specificity	Youden Index	95% CI
Salivary Calprotectin	8.57 μg/mL	0.79	0.52	0.31	0.12 – 0.48
Salivary Anti-CCP	28.54 Units	0.62	0.84	0.46	0.29 – 0.61

Note: CI, confidence interval. Youden Index calculated as (Sensitivity + Specificity - 1).

Supplementary Table S4. Logistic regression analysis and AUC of salivary Anti-CCP by quartiles.

Variable	Quartile	OR (95% CI) [Simple]	p-value	AUC (95% CI)	OR (95% CI) [Multiple*]	p-value	AUC (95% CI)
Salivary Anti-CCP	Q1 (Ref)	1	-	0.74 (0.65–0.83)	1	-	0.86 (0.79–0.93)
	Q2	0.74 (0.24–2.23)	0.588		0.46 (0.13–1.67)	0.237	
	Q3	1.16 (0.39–3.40)	0.791		0.88 (0.25–3.05)	0.835	
	Q4	25.35 (4.02–159.96)	<0.001		24.50 (3.57–167.99)	0.001	

* Adjusted for Age, Sex, Alcohol, Dry mouth, Hyperlipidemia

Supplementary Table S5. Variance Inflation Factor (VIF) analysis for variables used in logistic regression models.

Variable	VIF
Age	1.39638
BMI	1.28428
RF	1.13425
Anti_cpp	1.51459
ESR	3.09218
DAS28ESR	5.94791
DAS28CRP	3.01286
Salivary_Flow	1.31942
Salivary_CAL2	1.05495
salivary_anti_CCP	1.24457

* A VIF value < 8 indicates no significant multicollinearity.

Supplementary Table S6. Internal validation of logistic regression models using bootstrapping (1,000 iterations).

Model	Original AUC	Bootstrapped Mean AUC	95% CI	Standard Deviation (SD)
Model 1[a]	0.83	0.83	0.83 – 0.83	0.03
Model 3[b]	0.9	0.91	0.91 – 0.92	0.02

Abbreviations: AUC, area under the curve; CI, confidence interval. [a] Model 1 includes salivary calprotectin and anti-CCP. [b] Model 3 includes salivary calprotectin, anti-CCP, age, sex, alcohol exposure, dry mouth symptoms, and hyperlipidemia.

Supplementary Table S7. Sensitivity analysis of logistic regression models for salivary biomarkers stratified by alcohol exposure and hyperlipidemia.

Biomarker	Subgroup	Simple Model OR (95% CI)	*p*-value	Interaction *p*-value	Multiple Model* OR (95% CI)	*p*-value	Interaction *p*-value
Salivary Calprotectin	Non-drinker	1.08 (1.01–1.17)	0.033	0.371	1.15 (1.04–1.27)	0.005	0.591
	Drinker	1.15 (1.03–1.27)	0.01		1.19 (1.04–1.36)	0.013	
Salivary Anti-CCP	Non-drinker	1.07 (1.03–1.11)	0.001	0.063	1.09 (1.04–1.15)	0.001	0.026
	Drinker	1.01 (0.98–1.05)	0.444		1.01 (0.97–1.05)	0.793	
Salivary Calprotectin	hyperlipidemia Absent	1.13 (1.04–1.22)	0.002	0.857	1.18 (1.07–1.31)	0.001	0.711
	hyperlipidemia Present	1.14 (1.00–1.31)	0.052		1.14 (0.98–1.33)	0.099	
Salivary Anti-CCP	hyperlipidemia Absent	1.04 (1.01–1.07)	0.013	0.598	1.06 (1.02–1.10)	0.008	0.748
	hyperlipidemia Present	1.06 (1.00–1.11)	0.037		1.06 (1.01–1.12)	0.034	

* Note: OR, odds ratio; CI, confidence interval. For the analysis stratified by alcohol exposure, the multiple model was adjusted for Age, Sex, Alcohol, and Dry mouth. For the analysis stratified by hyperlipidemia, the multiple model was adjusted for Age, Sex, Dry mouth, and Hyperlipidemia.

Supplementary Table S8. Logistic regression analysis of salivary calprotectin stratified by dry mouth symptoms.

Subgroup	n (RA / HC)	OR (95% CI) [Simple]	p-value	Interaction p	OR (95% CI) [Multiple*]	p-value	Interaction p
No Dry Mouth	89 (42 / 47)	1.10 (1.04-1.17)	0.0019	0.8655	1.12 (1.04–1.20)	0.002	0.669
Dry Mouth	19 (16 / 3)	1.12 (0.90-1.39)	0.2963		1.39 (0.85–2.28)	0.186	

* Adjusted for Age, Sex, Alcohol, Dry mouth, Hyperlipidemia

Supplementary Table S9. Logistic regression analysis and ROC performance in an age-matched subset.

Variable	OR (95% CI) [Simple]	p-value	AUC (95% CI)	OR (95% CI) [Multiple*]	p-value	AUC (95% CI)
Salivary Calprotectin	1.14 (1.05–1.25)	0.004	0.75 (0.62–0.88)	1.55 (1.16–2.07)	0.003	0.93 (0.87–1.00)
Salivary Anti-CCP	1.05 (1.02–1.09)	0.004	0.74 (0.61–0.88)	1.07 (1.02–1.13)	0.007	0.87 (0.77–0.96)

* Adjusted for Age, Sex, Alcohol, Dry mouth, hyperlipidemia

Supplementary Table S10. Multivariate logistic regression analysis adjusting for Erythrocyte Sedimentation Rate (ESR) to assess the independence of salivary biomarkers.

Variable	Model	OR	95% CI	p-value
Salivary Calprotectin	Simple Model	1.11	1.05 – 1.18	<0.001
	Model 3*	1.14	1.06 – 1.23	<0.001
	Model 3 + ESR	1.35	1.08 – 1.68	0.008
Salivary Anti-CCP	Simple Model	1.05	1.02 – 1.08	<0.001
	Model 3*	1.06	1.02 – 1.09	<0.001
	Model 3 + ESR	1.05	1.00 – 1.10	0.074

* Model 3 adjusted for: Age, Sex, Alcohol, Dry mouth, Hyperlipidemia.

Supplementary Table S11. Comparison of salivary biomarker levels in patients with RA according to medication

Variable	Subgroup	N	Salivary Calprotectin (μg/mL) Median (IQR)	p-value*	Salivary Anti-CCP (Units) Median (IQR)	p-value*
Glucocorticoids	Users	36	12.28 (IQR)	0.111	51.10 (IQR)	0.261
	Non-users	22	17.95 (IQR)		29.87 (IQR)	
cDMARDs	Users	54	13.72 (IQR)	0.941	42.52 (IQR)	0.965
	Non-users	4	14.78 (IQR)		85.80 (IQR)	
Biologics	Users	12	10.58 (IQR)	0.265	23.14 (IQR)	0.166
	Non-users	46	14.37 (IQR)		47.92 (IQR)	
NSAIDs	Users	33	12.70 (IQR)	0.295	40.63 (IQR)	0.779
	Non-users	25	14.57 (IQR)		44.42 (IQR)	

* Mann–Whitney U test. Abbreviations: cDMARDs, conventional disease-modifying antirheumatic drugs; NSAIDs, non-steroidal anti-inflammatory drugs; IQR, interquartile range; Anti-CCP, anti-cyclic citrullinated peptide

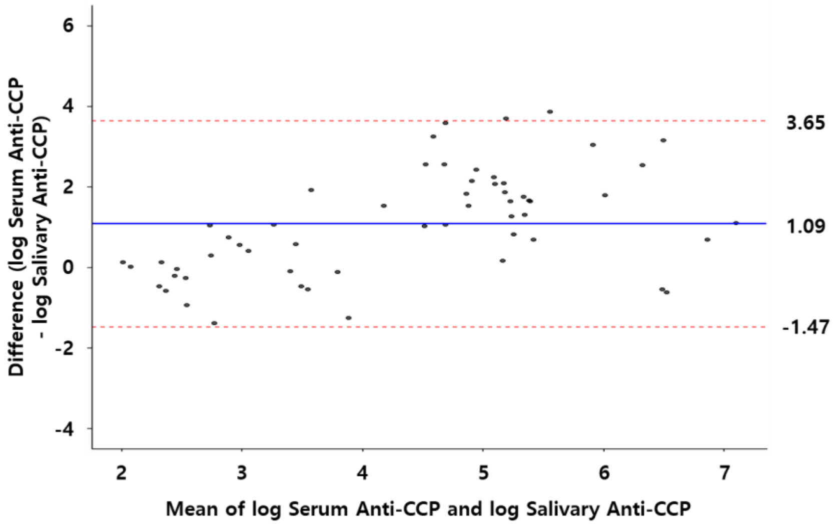

Supplementary Figure S1. Bland–Altman plot of agreement between serum and salivary anti-CCP levels. The plot displays the difference between log-transformed serum and salivary anti-CCP levels against their mean. The solid blue line represents the mean difference (bias = 1.09), indicating systematically higher levels in serum. The dashed red lines represent the 95% limits of agreement (–1.47 to 3.65).
